# Minimally Invasive Management of Zinner's Syndrome with Same-Session Robot-Assisted Seminal Vesiculectomy and Ipsilateral Nephroureterectomy Using a Single Geometry of Trocars

**DOI:** 10.1089/cren.2018.0066

**Published:** 2018-11-07

**Authors:** Murat Can Kiremit, Omer Acar, Alan Alper Sag, Ersin Koseoglu, Mert Kilic, Yakup Kordan, Mevlana Derya Balbay

**Affiliations:** ^1^ Department of Urology, Koc University Hospital, Istanbul, Turkey.; ^2^ Department of Urology, Koc University, Istanbul, Turkey.; ^3^ Division of Interventional Radiology, Department of Radiology, Eastern Virginia School of Medicine, Norfolk, Virginia.; ^4^ Department of Urology, Amerikan Hospital, Istanbul, Turkey.

**Keywords:** seminal vesicle cyst, renal agenesis, robot-assisted laparoscopic nephroureterectomy, ureteral ectopia, Zinner's syndrome

## Abstract

***Background:*** Seminal vesicle cyst is an extremely rare condition, which is frequently congenital and associated with Zinner's syndrome. This syndrome represents a constellation of seminal vesicle cyst, ipsilateral or contralateral renal agenesis or renal dysplasia, ureteral ectopia, and ejaculatory duct obstruction. We report a young symptomatic patient undergoing robot-assisted laparoscopic excision of a huge seminal vesicle cyst during which an atrophic ipsilateral kidney was discovered incidentally and managed by nephroureterectomy in the same session without changing trocar positions.

***Case Presentation:*** A 23-year-old male patient presented with a 2-year history of lower urinary tract symptoms, perineal pain, and recurrent urinary tract infections. Ultrasonography revealed the absence of left kidney and a fluid-filled cystic lesion located behind the bladder on the left side, which was consistent with cystic dilatation of the left seminal vesicle. MRI confirmed the diagnosis of a huge cystic structure originating from the left seminal vesicle and identified the presence of a rudimentary left ureter without an associated renal unit. Cystoscopy revealed bulging of the bladder neck at 6 o'clock position and the ureteral orifices at normal positions and configurations. Based on these findings, the clinical diagnosis was established as Zinner's syndrome. The present case was performed by Da Vinci Si robotic platform using the 5-trocar technique.

***Conclusion:*** Robot-assisted laparoscopic excision is a safe and feasible option to treat large seminal vesicle cysts, which may be a component of Zinner's syndrome. Simultaneous upper urinary tract interventions, such as nephroureterectomy, can be employed by redocking the robot and repositioning the patient, using the same layout of robotic trocars.

## Introduction

Seminal vesicle cyst is an extremely rare condition, which is frequently congenital and associated with Zinner's syndrome. This syndrome represents a constellation of seminal vesicle cyst, ipsilateral or contralateral renal agenesis or renal dysplasia, ureteral ectopia, and ejaculatory duct obstruction. It is caused by developmental malformations involving the distal portion of the mesonephric duct. Although seminal vesicle cysts are usually asymptomatic, they may be associated with or lead to lower urinary tract symptoms, perineal pain, ejaculatory disorders such as painful ejaculation or hematospermia, and infertility.

Depending on the presence and severity of accompanying symptoms, cysts originating from seminal vesicles may require interventional treatment, which may be employed through natural orifices (transurethral resection and transrectal aspiration), through open approach or by laparoscopic surgery. Seminal vesicle cysts tend to recur after transurethral and transrectal treatments.

Open surgical excision should be reserved for complex and/or recurrent cases in the era of minimally invasive laparoscopic surgery, which has gained substantial popularity and acceptance in a wide variety of urologic procedures owing to its advantages regarding postoperative pain, length of hospitalization, time to return to daily activities, and so on. Since its introduction to the urologic armamentarium, several authors have reported favorable results regarding the feasibility of laparoscopic management of seminal vesicle cysts.^1^ Subsequently and not surprisingly, robot-assisted laparoscopic excision has evolved as another valid option to treat this rare entity.

In this study, we report a young symptomatic patient undergoing robot-assisted laparoscopic excision of a huge seminal vesicle cyst during which an atrophic ipsilateral kidney was discovered incidentally and managed by nephroureterectomy in the same session without changing trocar positions.

Written informed consent was obtained from the patient.

## Presentation of Case

A 23-year-old male patient presented with a 2-year history of lower urinary tract symptoms, perineal pain, and recurrent urinary tract infections. The abdominal examination findings were unremarkable. Both testicles and vasa deferentia were palpable in the scrotum. Urinalysis parameters and serum creatinine level were within normal limits. Ultrasonography revealed the absence of left kidney and a fluid-filled cystic lesion located behind the bladder on the left side, which was consistent with cystic dilatation of the left seminal vesicle.

**FIG. 1.**
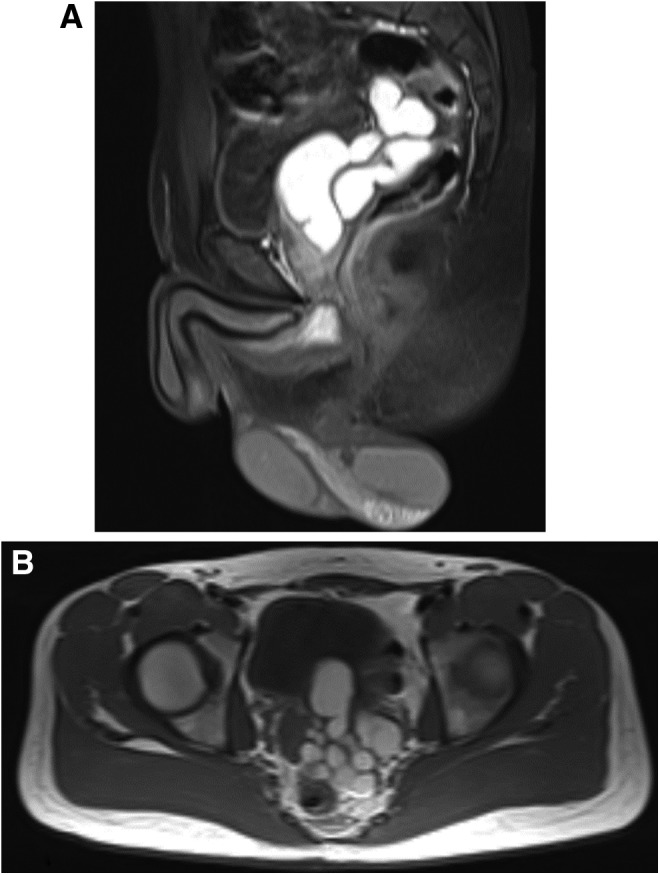
Sagittal **(A)** and axial **(B)** MRI images show a large (∼5 × 6.5 × 8 cm) tubular fluid-filled structure originating from the left seminal vesicle consistent with seminal vesicle cyst. A small left ureteral stump is evident, consistent with rudimentary ureter.

MRI confirmed the diagnosis of a huge cystic structure originating from the left seminal vesicle and identified the presence of a rudimentary left ureter without an associated renal unit (Fig. 1A, B). On uroflowmetry, urine flow was weaker than expected for his age with the maximal rate being 16 mL/second. Postvoid residual urine amount was negligible. Cystoscopy revealed bulging of the bladder neck at 6 o'clock position and the ureteral orifices at normal positions and configurations. Based on these findings, the clinical diagnosis was established as Zinner's syndrome.

## Robotic Surgery Technique

**FIG. 2.**
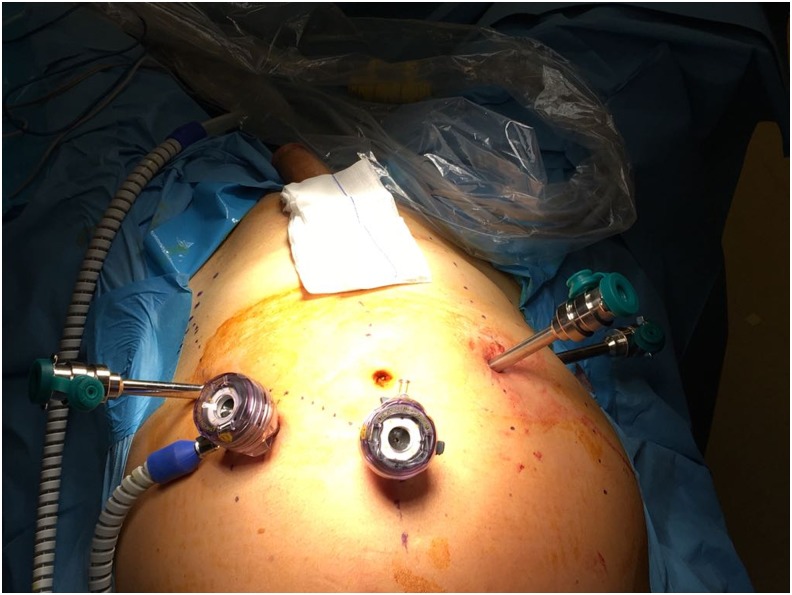
Five trochar technique. A 12-mm camera trochar, a 12-mm bedside assistant trochar, and three additional 8-mm trochars.

The patient was placed in Trendelenburg position and a Foley catheter was inserted into the bladder. Veress needle was introduced through the supraumbilical incision, and the abdomen was insufflated with CO_2_ to 12 mm Hg. Then the Veress needle was replaced with a 12-mm trocar through which a 10-mm 0° lens was inserted. Three additional 8-mm trocars and one 12-mm bedside assistant trocar were placed lateral to the rectus musculature bilaterally, right lower quadrant and between the left trocar and the camera trocar, respectively (Fig. 2).

The peritoneum overlying the superior aspect of the dilated left seminal vesicle was incised using shears and reflected superiorly. The left vas deferens was mobilized to retract the left ampulla and seminal vesicle medially. Care was given not to injure the pararectal plexus located lateral to the tips of the seminal vesicle while dissecting it off. The left seminal vesicle was then transected at its base using a shear and applying clips after all fascial attachments were freed. Right vas deferens and seminal vesicle were left untouched during the dissection to preserve fertility. The specimen was removed intact through the 12-mm port using a specimen retrieval bag. During dissection of the left vas deferens, the left ureter was identified and followed to the level of common iliac vessels. However, orientation of the docked robot and its arms hindered further cranial dissection.

Therefore, to dissect and mobilize the left ureter along its entirety, robot was undocked while leaving the trocar positions unchanged. After placing the patient in a lateral decubitus position, robot was redocked from the left side of the patient. These manuevers took 20 minutes. As the left ureter was dissected up to the renal hilum, dysplastic renal tissue and its corresponding vasculature were encountered. The renal pedicle was controlled by applying hem-o-lock clips and nephroureterectomy was completed after dissecting perinephric fat tissue. The specimen was removed through the 12-mm port in a retrieval bag.

The duration of the procedure was 145 minutes and the estimated blood loss amount was 40 mL. Foley catheter and drain were removed on the first and second postoperative days, respectively. The patient was discharged on postoperative day three after an uneventful hospital course. He did not report any symptoms on his follow-up visit at the third month of the surgery. Pathological examination did not identify malignant elements in the excised specimens.

## Discussion and Literature Review

Zinner's syndrome is a rare congenital Wolffian duct anomaly, which was first described in 1914.^2^ It is considered as the male counterpart of the Mayer–Rokitansky–Kuster–Hauser syndrome, which is characterized by the deficiency of Müllerian duct-derived female internal genital organs. Patients diagnosed with Zinner's syndrome are usually asymptomatic, but a wide range of symptoms (abdominal/pelvic pain, lower urinary tract symptoms, ejaculation disorders, and fertility issues) may be present clinically. Surgical excision of the seminal vesicle cyst(s) may be advocated in symptomatic patients because other treatment alternatives such as transrectal aspiration or transurethral unroofing have a high recurrence rate and may lead to infectious complications.^3^

Historically, open surgery was the gold standard treatment for large seminal vesicle cysts. However, contemporary practice would mainly rely on laparoscopic or robotic surgery, considering the greater likelihood of complications such as rectal, bladder, and ureteral injuries, pelvic urinoma, and erectile dysfunction associated with the open approach, which is often conducted in a non-nerve sparing manner. Widespread adoption and growing level of experience in robotic radical prostatectomy have enabled urologists to offer and employ robotic surgery to manage nononcologic pelvic disorders, such as seminal vesicle cysts, in a safe and feasible manner. This effectiveness can be attributed to the fact that robot-assisted laparoscopic exposure provides direct access to the seminal vesicles and magnified three-dimensional image input for the deep retrovesical region.

The present case was performed by Da Vinci Si robotic platform using the 5-trocar technique, as described earlier (Fig. 2). When camera is introduced from above targeting distal pelvic structures, its tip cannot be directed to observe cranial structures since it clashes with the robotic arm. This limitation prevented us to follow and see the ureteral remnant and its accompanying dysplastic renal parenchyma located cranially. This limitation was alleviated when the position of the patient was changed from Trendelenburg to lateral decubitus, where camera was inserted from the same trocar but directed laterally to observe more cranial structures. Proximal regions along the iliac vessels and paracolic structures were easily be seen and followed with the camera when the patient was in this position.

Our primary aim was to preserve the minimally invasive nature of the surgery. Therefore, we tried to avoid using additional trocars/ports. If patient repositioning and robot redocking have not provided optimal exposure for an efficient excision, then we would have to make use of additional trocars.

From a uroradiologic perspective, Zinner's syndrome is challenging because it is rare, the findings are often considered “incidental” in the absence of convincing clinical correlative symptoms, and the renal and seminal vesicle findings span two radiologic “zones”: the “abdomen” and the “pelvis,” respectively (which are not always imaged together). Moreover, these patients are young and may have a functionally solitary kidney, which provides impetus to avoid ionizing radiation and iodinated contrast media, respectively. Goals of imaging should be to exclude obvious malignancy (invasive lesion and lymphadenopathy), to identify the location of the seminal vesicle cyst for surgical planning (median, paramedian, and lateral), and to grossly evaluate renal presence or atrophy, as dysplastic renal tissue can be premalignant and this affects surgical approach and informed consent of the patient.

Retroperitoneal ultrasonography may not reliably characterize the dysplastic histopathologic features of the affected renal unit. Ultrasonography of the pelvis (not transrectal) might fail to identify the pelvic cyst, depending on cyst size, distribution of bowel gas at the time of the scan and the patient's ability to cooperate with the sonographer for positioning and maintaining a full bladder. Despite these limitations, ultrasonography is typically the first-line imaging study because it is inexpensive, widely accessible, and does not involve ionizing radiation or iodinated contrast material.

MRI is often the next step in younger patients (as in this case). If the MRI is prompted by a pelvic finding, the MRI scan region will usually include only the pelvis (and not the abdomen). Still, careful review of the provided initial localizer images may reveal or confirm incidental renal agenesis or atrophy.

Gadolinium is preferable and generally provided when neoplasm is in the differential. Routine MRI protocols allow evaluation for differential considerations, which typically include prostatic duct-utricle cyst, prostate abscess, bladder diverticulum, ectopic ureterocele, and ectopic pelvic hydronephrotic kidney. A detailed review of imaging findings is beyond the scope of this report but readily available in the literature.^4^

This report is unique in that urologic rescue measures were needed during the operation, as the ipsilateral atrophic kidney was not identified at preoperative imaging. The robotic surgical plan was adjusted during the surgery to accommodate for real-time findings. It is possible that the findings were not considered noteworthy for the report before imaging because the disease is rare; however, at some institutions it is also possible that the localizer images for the MRI were not sent by the technologist or not reviewed by the uroradiologist, as they are often of lower resolution. Another chance to suggest renal atrophy from just a pelvic MRI would be with ureteral asymmetry; however, even this is often attributed to ureteral peristalsis at MR urography.

Therefore, in the correct age group with seminal vesicle cysts, it may behoove the uroradiologist to maintain a high index of suspicion for upper urinary tract anomalies and recommend completion imaging of the abdomen where appropriate. Similarly, in countries where first-line CT is more prevalent, unexplained renal atrophy may prompt further evaluation for lower urinary tract anomalies that may explain the patient's symptoms.

## Conclusion

Robot-assisted laparoscopic excision is a safe and feasible option to treat large seminal vesicle cysts, which may be a component of Zinner's syndrome. Simultaneous upper urinary tract interventions, such as nephroureterectomy, can be employed by redocking the robot and repositioning the patient, using the same layout of robotic trocars. Excellent exposure of the retrovesical and retroperitoneal area together with the well-known advantages of laparoscopic surgery such as minimal blood loss, short hospital stay, and a rapid overall recovery asserts the robotic approach as a more preferable procedure when planning extirpative surgery in pelvic cavity.

## Abbreviations Used

CT: computed tomography
MRI: magnetic resonance imaging

## References

[1] Cherullo EE, Meraney AM, Bernstein LH, Einstein DM, Thomas AJ, Gill IS Laparoscopic management of congenital seminal vesicle cysts associated with ipsilateral renal agenesis. J Urol 2002;167:1263–1267 11832710

[2] Zinner A Ein fall von intravesikaler samenblasenzyste. Weien Med Wschr 1914;64:604–610

[3] Patel B, Gujral S, Jefferson K, Evans S, Persad R Seminal vesicle cysts and associated anomalies. BJU Int 2002;90:265–271 12133063 10.1046/j.1464-410x.2002.02883.x

[4] Livingston L, Larsen CR Seminal vesicle cyst with ipsilateral renal agenesis. Am J Roentgenol 2000;175:177–180 10882270 10.2214/ajr.175.1.1750177

